# Intersecting inequalities, gender and adolescent health in Ethiopia

**DOI:** 10.1186/s12939-020-01214-3

**Published:** 2020-06-15

**Authors:** Nicola Jones, Kate Pincock, Sarah Baird, Workneh Yadete, Joan Hamory Hicks

**Affiliations:** 1 Gender and Adolescence: Global Evidence Research Programme and ODI, London, UK; 2 Gender and Adolescence: Global Evidence Research Programme and Refugee Studies Centre, University of Oxford, Oxford, UK; 3 Gender and Adolescence: Global Evidence Research Programme and Department of Global Health and Economics, George Washington University, Washington, DC USA; 4 Gender and Adolescence: Global Evidence Research Programme, Addis Ababa, Ethiopia; 5 Gender and Adolescence: Global Evidence Research Programme and Department of Economics, University of Oklahoma, Norman, USA

**Keywords:** Intersectionality, Health, Adolescence, Gender, Inequality

## Abstract

**Background:**

Until recently, global public health initiatives have tended to overlook the ways that social factors shape adolescent health, and particularly how these dynamics affect the specific needs of adolescents in relation to information about puberty, menstruation and sexual health. This article draws on mixed methods data from rural and urban areas of Ethiopia to explore how access to health information and resources - and subsequently health outcomes - for adolescents are mediated by gender and age norms, living in different geographical locations, poverty, disability and migration.

**Methods:**

Data was collected in 2017–2018 for the Gender and Adolescence: Global Evidence (GAGE) mixed-methods longitudinal research baseline in three regions of Ethiopia (Afar, Amhara and Oromia). Quantitative data was collected from over 6800 adolescents and their caregivers, with qualitative data obtained from a sub-sample of 220 adolescents, their families and communities. Adolescent participants shared their experiences of health, illness and nutrition over the previous year; their knowledge and sources of information about sexual and reproductive health and puberty; and their attitudes toward sexual and reproductive health. Regression analysis was used to explore differences by gender, age, rural/urban residence, and disability status, across a set of adolescents’ health knowledge and other outcomes in the quantitative data. Intersectional analysis was used in analysing the qualitative data.

**Results:**

Analysis suggested that gender inequality intersects with age, disability and rural/urban differences to shape young people’s access to information about puberty, with knowledge about this topic particularly lacking amongst younger adolescents in rural areas. Drought and lack of access to clean water exacerbates health challenges for adolescents in rural areas, where a lack of information and absence of access to preventive healthcare services can lead to permanent disability. The research also found that gaps in both school-based and alternative sources of education about puberty and menstruation reinforce stigma and misinformation, especially in rural areas where adolescents have higher school attrition rates. Gendered cultural norms that place high value on marriage and motherhood generate barriers to contraceptive use, particularly in certain rural communities.

**Conclusions:**

As they progress through adolescence, young people’s overall health and access to information about their changing bodies is heavily shaped by intersecting social identities. Structural disadvantages such as poverty, distress migration and differential access to healthcare intersect with gender norms to generate further inequalities in adolescent girls’ and boys’ health outcomes.

## Background

### Introduction

Adolescence is a critical life stage characterised by significant emotional and physical changes, during which patterns of behaviour and development lay the foundations for health outcomes later in life. The global adolescent population stands at more than 1.2 billion, most of whom live in the Global South. In 2017, the Lancet Commission on adolescent health and wellbeing suggested that ‘*co-ordinated investments in adolescent health and wellbeing are among the best investments that can be made by the human community to achieve the UN’s Sustainable Development Goals’* [1]. Yet as the Commission also highlights, until very recently, global public health initiatives have overlooked the specific needs of adolescents and young adults, leading them to be ‘left behind’ [2] within health policies and programming.

The 2030 Sustainable Development agenda, especially Goal 3 on health and well-being, and Goal 5 on gender equality, has particular relevance for addressing these gaps in policy and programming. The Sustainable Development Goals also emphasize that addressing structural drivers of socioeconomic inequalities is key to achieving the goal of ‘leave no-one behind’ [3]. Ethiopia is a strong case study for exploring these issues. It is a lower-income country which has made significant investments in health over the last two decades, and the government’s system of ‘health extension workers’ is held up in Africa as a model of good practice. Yet despite efforts to expand ‘youth friendly programming’ through the National Adolescent and Youth Reproductive Health Strategy (2016–2020), health programming in Ethiopia still pays limited attention to gender and other dynamics of diversity which produce marginality for some adolescents in relation to health.

In order to achieve the Sustainable Development Goals objective of leaving no-one behind, inequalities in health outcomes must be addressed. The purpose of this paper is to thus explore how access to health information and resources - and subsequently health outcomes - for adolescents are mediated by gender and age norms, living in different geographical locations, poverty, disability and migration, with an aim of improving policy and programming around adolescent health. We first reflect on the key challenges for adolescent health in the global South and in Ethiopia specifically, before drawing on mixed-methods approach and intersectional analysis to show that poverty, distress migration and differential access to healthcare intersect with gender norms to generate further inequalities in adolescent girls’ and boys’ health outcomes.

### Adolescent health challenges

Adolescence brings far-reaching changes, including new health challenges linked to the onset of sexual activity, emotional control and behaviour [4, 5]. How these biological changes are interpreted socially has a significant impact on the services available to adolescents, the information they have access to (in the home and from peers), and their ability to access timely and appropriate healthcare. Inequalities in health and wellbeing are differences in health within a population that stem from discrimination or inaccessibility of certain resources; poor health outcomes are often experienced by those who are disadvantaged on other bases, such as age, gender, disability or place of residence [6]. There is broad recognition that biological differences play an important role in health inequalities between adolescent boys and girls, for example in adolescent girls’ greater susceptibility to HIV [7]. However, there is now a growing consensus that most inequalities in adolescent health are social in nature: the emotional and physical transformations adolescents undergo change how their families, communities and societies regard them [2]. The social norms and attitudes of communities are thus of key importance.

Whilst boys and girls experience differences in general health, nutrition, and sexual and reproductive health, these do not always mean that girls are the most disadvantaged in all areas [8]. Global research shows that girls are more likely to report poor health outcomes, including poor mental health [9, 10]; but boys are more likely to engage in substance use and commit suicide [11, 12]. The health and nutrition of adolescent boys is often prioritised in Ethiopia when it comes to intrahousehold hierarchy because of their role in livelihood security [13]. Despite efforts to expand adolescents’ access to sexual and reproductive health through the Ethiopian government’s health extension workers’ scheme, and ‘youth friendly programming’ including the National Adolescent and Youth Reproductive Health Strategy (2016–2020), girls’ domestic workload at home and the stricter rules they are expected to adhere to that confine them to the home even when they are not working [14]. Negative attitudes towards adolescents’ sexuality from health extension workers also deters adolescents from using youth-friendly reproductive health services [15].

This body of work shows that gender norms and attitudes are a key social determinant of adolescents’ health [16–18]. However, many other factors also shape access to health information, resources and outcomes. It is known that gender intersects with structural inequalities such as rural/urban location, economic status, educational attainment, and geopolitics across different regions and states in Ethiopia [8]. Looking only at gender in this context therefore obscures the multidimensionality of poverty and the reproduction of inequality and disadvantage within particular communities. For example, whilst research has found that girls aged 13–17 are more likely to report food insecurity than boys, prevalence is much higher in rural areas where food is scarce than in cities, indicating that poverty exacerbates gender-unequal outcomes [19]. The evidence on adolescents with disabilities is extremely fragmented, especially in the global South [20, 21] yet there is a growing recognition that age, disability and gender intersect in complex ways, with girls more disadvantaged by disability than their male peers due to the confluence of restrictive gender norms and disability-related stigma [22, 23] The evidence base on Ethiopian adolescents’ physical health also primarily focuses on health needs and behaviours of adolescents over the age of 15, with the potential challenges facing younger adolescents largely ignored.

Feminist researchers have raised concerns that with gender equality becoming a key objective for global development, gender mainstreaming in health policy is currently implemented with a simplistic and reductive understanding of gender inequality [24]. Work linking gender and health is based on assumptions about the primacy of gender inequality above other social inequalities [25], and often fails to address the nuance and diversity of gendered experiences [26]. Calls for an approach which engages with the underlying social and structural drivers of health-related issues – such as poverty, rural/urban divides and vulnerability to disease - facing young people in the Global South have been increasingly issued by those working in the field of adolescent health [2]. In attending to the Sustainable Development Goals’ call to ‘leave no one behind’, paying due attention to adolescents’ experiences of social and structural inequalities and the ways that these shape access to health information and resources is key for understanding what factors are driving inequalities in outcomes for adolescent boys and girls - and informing interventions to address them. This paper thus contributes to a growing body of research that addresses the ways that social and structural inequalities reinforce marginality in health-related knowledge and outcomes for adolescent girls in Ethiopia, focusing specifically on four dimensions of health: general health, nutrition, knowledge about puberty, and knowledge of and access to contraception.

## Conceptual framing and methods

### Conceptual framing

Academic research into the practical challenges facing young people growing up in contexts of poverty and inequality has often overlooked the specific vulnerabilities and challenges associated with adolescence as a stage in the lifecycle in favour of a gender lens. The analysis of data presented here draws on the capabilities approach and intersectional theory, which taken together provide an alternative paradigm to a framing that centres only on gender, encompassing other dimensions of social identity and the ways they interact. The capabilities approach pioneered by Amartya Sen [27, 28] and developed by Martha Nussbaum [29] asserts that being able to achieve outcomes such as good overall health, nutrition, and knowledge about puberty and contraception is contingent on more than just healthcare and information being available; these resources must be accessible to all those who need them. As suggested in the existing literature, being able to access health information and resources is shaped by gender, but also by age, ethnicity, poverty, disability and a host of other social dynamics; the capabilities approach emphasizes the need to attend to the obstacles these present in promoting wellbeing.

The GAGE conceptual framework adopts a capabilities approach to emphasize that investments in adolescents must support their development in all areas of life. To this end, six capability ‘domains’ are identified within the GAGE conceptual framework as areas where adolescents need particular support and services in order to achieve positive outcomes. These are education and learning; bodily integrity and freedom from violence; voice and agency; psychosocial wellbeing; economic empowerment; and health, nutrition and sexual and reproductive health. It is this last domain that the findings in this paper explores; this domain is concerned with adolescent knowledge and awareness about their bodies and how to maintain good nutrition and keep healthy, including access to the knowledge, supplies and services needed to manage menstruation and protect themselves from sexually transmitted infections and unplanned pregnancy [30].

An intersectional approach complements the focus on capabilities by attending to the mutually constitutive and relational role of social identities [31] in enabling or constraining the freedom of adolescents to access the nutrition and health knowledge, resources and outcomes that are necessary for them to have a good quality of life [28]. An intersectional approach rejects the idea that ‘gender’ as a single category should be central to analysis, instead emphasising the ways in which inequalities including age, disability, socioeconomic status, rurality and other social categories interact with and co-constitute each other to affect outcomes such as health [32]. Originating with black feminist scholars [33–35], intersectionality emphasises the ways in which different aspects of social identity converge to produce particular experiences of marginalisation. These structures and processes are dynamic, shifting across time and place [36–38]. Because health inequalities stem from discrimination, adolescents who are already socially and economically marginalised, including ethnic minorities, refugees and indigenous adolescents, and adolescents with disabilities, have the most to lose out when these dimensions are not taken into account.

The analysis for this study thus focuses on the ways that different constraints on adolescents’ capabilities intersect to mutually reinforce each other. Data on adolescents’ sex, age, disability status, residential location and everyday lives was complemented by information from adult caregivers on household, family background, dwelling characteristics, access to credit, role in household decision making, and attitudes and social norms. In addition, community and school surveys included sections on community characteristics, schools, health facilities, religion and gender practices [39].

This article draws on baseline data from GAGE (Gender and Adolescence: Global Evidence) – a longitudinal mixed-methods research study exploring what works to support the development of adolescents’ capabilities during the second decade of life (10–19 years). The baseline research explored the patterning of adolescent girls’ and boys’ experiences in Ethiopia as they transition from early adolescence through puberty and into early adulthood. Data was collected between late 2017 and early 2018, including both quantitative surveys with more than 6800 adolescents and their caregivers, and qualitative data from a sub-sample of over 220 adolescents, their families and 33 key informant interviews at district (6) and community (27) levels including teachers, social workers and health workers. The quantitative research was generated through face-to-face administration of survey questionnaires, and the qualitative data through a variety of interactive individual and group in-depth qualitative tools, including focus groups, key informant interviews and participatory activities such as body mapping and community and institutional mapping. The purpose of using these modes of qualitative research in addition to a survey approach was to add detail and nuance to the quantitative data, and provide space for participants’ voices and experiences to come through in their own words when it came to understanding how inequalities affected their health in different ways.

### Data collection

Ethical review and permission for this research was obtained through the ODI Research Ethics Committee, the Addis Ababa University College of Health Sciences Institutional Review Board, and George Washington University Committee on Human Research, Institutional Review Board. Researchers were trained on how to appropriately and responsively interact with adolescents in exploring sensitive issues. Verbal assent to participate in the research was obtained from adolescents under the age of 18, and verbal consent was obtained from their caregivers. Surveys were translated into Afaan Oromo and Amharic, depending on the region, and were piloted and tested before being undertaken across the sample. Data was generated through face-to-face interviews with researchers from the same region as and of the same sex as the adolescent. The quantitative interviews were conducted in people’s homes, while the qualitative interviews and group activities were predominantly carried out at local schools, except in cases where respondents had limited mobility, for example, due to disability. The quantitative interviews were checked for internal consistency and completeness. The qualitative interviews were recorded and transcribed.

The sample included two cohorts: younger adolescents (aged 10–12 years, in urban, rural and pastoralist areas) and older adolescents (aged 15–17 years, only in urban areas). The research sites included communities in three regions of Ethiopia (Afar, Amhara and Oromia), and one city administration (Dire Dawa). Rural sites were selected for having high rates of child marriage (used as a proxy for conservative gender norms) and relative economic disadvantage. In each rural region there are five GAGE research sites (*woredas* or districts), comprising 175 communities (*kebeles*). The three urban GAGE sites were selected to explore the effects of urbanicity on adolescence; and to provide a point of comparison with rural sites to which they are geographically close, to help capture rural–urban migration. Given the cultural diversity of Ethiopia, the range of site selection also makes it possible to compare urban and rural differences in the context of similar socio-cultural environments. A household census was used to identify potential respondents in the sites, and a random sample of female and male adolescents was drawn from this census for the quantitative research. In addition, researchers purposively sought out-of-school adolescents and adolescents with disabilities to include in the research. The sample chosen for qualitative interviews was a subset of this larger quantitative sample. For sample overviews, see Tables 1 and 2.

**Table 1:** Quantitative sample of adolescents by gender, age, and geographic location

	# Observations
Total	6822
Young (aged 10–12)	5491
Older (aged 15–17)	1331
Male	3058
Female	3764
Rural	4645
Urban	2177

**Table 2:** Qualitative sample of adolescents and parents by gender and geographic location

	Nodal Girls			Mothers		Nodal Boys			Fathers			
	10–12	15–17	Sum			10–12	15–17	Sum			Total adolescents	Total parents
RURAL	61	29	90	**74**		60	22	**82**	**53**		**172**	**127**
URBAN	18	30	**48**	**39**		16	27	**43**	**29**		**91**	**77**
**TOTAL**	**99**	**59**	**158**	**118**		**76**	**49**	**125**	**82**		**266**	**204**

### Data analysis

Analysis of the quantitative survey data focused on a set of indicators related to the health capability domain, including self-reported health, experience of serious illness or injury in the past 12 months and whether treatment had been sought, food security experience, height-for-weight z-scores, BMI for age z-scores, existence of a source of information about puberty, activities affected by menstruation, ability to name a modern method of contraception, and attitudes related to sexual and reproductive health. Summary statistics describing these indicators are presented, with differences explored across gender, age, residential location, and disability status using regression analysis, The regressions are weighted to reflect the probability of being included in the study sample, and thus provide results that are representative of the study areas. Standard errors are clustered by community. Here we report only differences that are statistically significant at the *p* < 0.05 level.

For the qualitative research component, preliminary analysis took place during daily and site-wide debriefings with the research team, with findings informing the development of the thematic codebook. Following data collection, all interviews were transcribed and translated by native speakers of the local language, then coded using the qualitative software analysis package MAXQDA. The codebook was shaped around the GAGE conceptual framework of ‘3 Cs’ (capabilities, change strategies, and contexts) (GAGE consortium, 2019) but allowed flexibility to incorporate local specificities. The tools used for this analysis included individual object-based interviews which allowed adolescents to focus on issues that were meaningful to them, group-based body mapping exercises which explore adolescent transitions, and a community social norms mapping exercise with parents of adolescents to understand age- and gender-based norms and how they affect young people in their community, including change over time.

In terms of mixed methods analysis, we employed an iterative process, with the qualitative team drawing on the narratives generated by adolescents, parents and community key informants to sense make the quantitative findings, and then the quantitative team delving further into disaggregating data to explore emerging patterns within and across sites. This iterative process was particularly important in the case of discussions on sensitive issues related to for example norms on sexual and reproductive health, violence and harmful traditional practices.

## Results

GAGE data shows that rural/urban inequalities lead to unequal general health and nutrition outcomes for adolescents of both genders, and that disability amplifies these inequalities. With regards to knowledge about puberty, menstruation and contraception, stigma and social norms related to age and gender heavily shape access to information. Structural factors such as poverty, environmental degradation, and lack of services produce multiple disadvantages for adolescents already facing inequalities due to age, sex and (dis) ability and lead to unequal health knowledge and outcomes across all four dimensions assessed. GAGE’s mixed methods research gives a more nuanced picture than has previously been available from existing studies, highlighting the role of social norms and structural inequalities in shaping health and sexual and reproductive health outcomes.

### General health

General health among young adolescents was self-reported as good (88%). However, there was significant variation across urban/rural residential location, with those living in rural areas 4% less likely to report good health than their urban counterparts (see Tables 3 and 4 in Appendix). Our qualitative research findings suggested that adolescents in rural areas have a higher prevalence of sanitation-related illnesses than their urban peers. As a 12-year-old boy from South Gondar explained: ‘*I recently suffered from a stomach sickness. I got treatment and the health worker told me the sickness is because of unclean water’*. Poverty-related health inequalities are exacerbated for adolescents in rural areas by structural barriers including drought and lack of access to clean water; malaria and other infectious diseases due to lack of access to preventative or curative healthcare services; and preventable forms of disability caused both by lack of awareness and absence of services.

Over half (51%) of younger adolescents in the quantitative survey reported that they had experienced a common health symptom (e.g. vomiting, diarrhoea, coughing, fatigue) in the month preceding the interview. Significant accidents and illnesses were also relatively common findings of the quantitative survey: over the course of the past year, 16% reported that they had had a serious illness or injury. Many of these injuries are related to the environmental risks that adolescents face, including injury while herding cattle, travelling considerable distances to collect firewood or water, and illness or injuries to snake bites. These risks are particularly borne by adolescent boys; one young boy explained *‘Herders often exposed to water borne diseases as they don’t have access to clean water and they usually drink the available water in the ground.’*

Once again, there were substantial differences by residential location, however, with urban young adolescents much more likely (34%) to have experienced recent illness or injury. In rural lowland areas like East Hararghe, adults reported increased rates of malaria in qualitative interviews: ‘*Earlier there were no malarial diseases but now it started to be seen in our village...*’ Some permanent disabilities are the result of lack of access to timely healthcare for treatable illnesses (such as blindness caused by trachoma). However, in urban areas, adolescents were found to be at greater risk of road traffic accidents. The quantitative data shows urban young adolescents were 25% more likely to seek treatment than rural adolescents if experiencing a health issue.

Disability emerged as a critical intersecting factor within the qualitative findings. In qualitative interviews some adolescents with disabilities explained that permanent impairments had been the result of accidents and untreated illnesses. A girl with a mobility impairment in Community B, Zone 5 (Afar), for example, was maimed by a crocodile in the river that the community use for bathing. Another girl from Amhara was left blind by trachoma (a preventable and curable eye infection) that went untreated. The quantitative survey findings in turn highlighted that adolescents with disabilities enjoy significantly poorer health overall than their peers without disabilities. Only 44% of younger adolescents with disabilities reported being in good health in contrast to 89% of those without disabilities. Moreover, among younger adolescents in the quantitative survey, those with disabilities were more likely to report recent health symptoms than those without (70% versus 51% and twice as many reporting having suffered from a serious illness or injury in the past year (33% versus 16%).

General health inequalities also vary by sex and age, particularly for the urban adolescents. Among those living in urban sites, younger adolescents reported significantly better general health than older adolescents, and boys reported better health than girls (for both older and younger age groups). That said, in the longer term, adolescent boys may face a variety of health risks from their relatively higher rates of alcohol and *khat*^1^ use (though substance abuse was reported to be on the rise across rural and urban locations in the quantitative survey). In urban areas, young people attributed this to peer pressure in qualitative interviews, whereas in rural locations, it was attributed to young seasonal migrants returning home having developed bad habits. In South Gondar, one adult commented that ‘*Young migrants are returning habituated with bad behaviours like addiction of drugs,* khat*, alcohol and cigarettes.’.*

### Nutrition and food insecurity

Geographical, gender, and age differences were also stark in terms of adolescent nutrition and exposure to food insecurity. The quantitative data suggests young adolescents from rural areas have 74% higher food insecurity than their urban counterparts, and while both rural and urban adolescents on average have a height-for-age z-score (HAZ) below zero, rural young adolescents are 77% more likely to be short for their age and 95% more likely to have a lower body mass index (BMI) than their urban peers – indicators which both suggest longer-term malnutrition and patterns of disadvantage since earlier childhood. The survey findings also indicate that younger adolescent girls on average have a lower HAZ than boys and that among urban adolescents, older adolescents have a lower HAZ than younger adolescents. Moreover, in order to cope with food insecurity adolescents also reported that both boys and girls in rural areas were more than twice as likely to have suffered from cut backs in food consumption at the household level, with rural girls at slightly higher risk than boys (27% compared to 28%).

Our qualitative research nuances these findings and emphasises differences in adolescents’ experiences of food insecurity, which is unsurprising in a country still largely dependent on subsistence agriculture and where ‘*production is different from one household to the other*’ (teacher, Community I, East Hararghe). In South Gondar in the north of Ethiopia, most adolescents reported that they had enough to eat and that their diets were relatively diverse. Many young people reported eating three meals a day, and some reported consuming foodstuffs including *injera* (traditional bread), *wat* (stew) and fruits and vegetables, both from home production and the market. By contrast, many of those in the east of the country in East Hararghe and pastoralist Afar reported going hungry. A 10-year-old boy in Community I (East Hararghe) noted that: ‘*The issue of food is the other thing that worries me as the sorghum we have on our farm is about to end. If it is over, we will get hungry as it is the only crop we have*’. Teachers and adolescents reported that many students are not able to pay attention in class because they are hungry. As a teacher in Community E (South Gondar) explained: ‘*There are many children who come to school without having breakfast*’, impacting their ability to learn. A 12-year-old girl in Community I (East Hararghe) confirmed this: ‘*I don’t think about anything else except the food. You lose interest in learning*’. Adolescents with disabilities also reported a 13% higher incidence of food insecurity than their non-disabled peers.

### Puberty and menstruation

Adolescents were found to have limited access to information, services and supplies to support them through puberty, and girls had limited support with menstrual management. Disadvantage was shaped by geography, schooling levels and age. Only 52% of younger adolescents in rural areas reported having a source of information about puberty in the quantitative survey. Moreover, according to qualitative interviews, the information they did have tended to be general rather than practical, with no specific mention of the physical changes boys and girls undergo. This is problematic for girls because it means they are typically unprepared for menstruation. In GAGE research sites, the quantitative survey found that only 47% of schools had classes on puberty, with most introducing these classes only for older girls.

The qualitative survey identified some of the ways that stigma prevents mothers from talking to their daughters about menstruation, and this lack of information from family sources contributes to girls’ anxiety and fear about what lies ahead. As one young adolescent girl in the rural area of East Shewa recalled: ‘*I saw the blood on my sister’s clothes, I thought it was HIV/AIDS!*’ When she asked her mother, she was warned not to tell anyone what she had seen, and that she would be beaten if she raised the issue again. Another older girl in South Gondar explained that she spent the whole day vomiting and in bed: ‘*I didn’t know it would be so painful … I was concerned that I had been exposed to another disease.*’ Moreover, many adolescent girls continue to be marginalised by gender norms that drive stigma and discomfort around menstruation, particularly for younger girls in rural Ethiopia.

There are also serious resource challenges around creating a supportive environment for girls during puberty. 26% of young adolescent females reported in the quantitative survey that their normal activities are impacted by menstruation. Limited school facilities and poor access to menstrual hygiene management products, as well as teasing by boys, deter girls from attending school during their periods. Only 29% of schools in GAGE research sites have running water, and only 14% have private rooms for girls to change their sanitary products.

The rural–urban divide intersects with gender inequality to shape young people’s access to information about puberty. Adolescents in urban areas are slightly better able to identify signs of puberty in boys and girls than their rural peers. Indeed, the quantitative survey found that many rural girls believe myths about menstruation that link it to sexual activity or signs of illness. Only half of the younger adolescents in rural areas had a source of information about puberty, compared to 64% of their urban counterparts. However, in the younger urban cohort, boys were significantly more likely to report having a source of information than girls (69% compared with 60%). While many adolescents reported getting information from the media, only urban adolescent girls reported receiving useful and accurate advice from family members. Age was also a factor, with 94% of urban older adolescents having a source of information. This means that many younger adolescents, when they begin puberty, are misinformed and have nowhere to turn for accurate information.

Disability status also intersected with age, gender and rural disadvantage. The quantitative survey found that adolescent girls with disabilities were 15% less likely to have a source of information about puberty, likely reflecting greater risks of being out of school and also having less access to out of school platforms that provide life skills information, including relating to puberty and sexual and reproductive health.

### Contraceptive knowledge, access and use

The research found that despite generally good physical availability of contraception in Ethiopia through its HEWs, there were significant differences by region. These were largely based on gendered cultural norms which were stronger in certain areas. Whereas 40% of younger adolescent girls in South Gondar could identify a method of contraception in the quantitative survey, only 14% in East Hararghe could do so and 7% in Afar Zone 5 (though some could describe contraceptive options without being able to name them). One out-of-school younger adolescent girl in a group body mapping exercise in rural East Hararghe explained: ‘*I know pills, implants that are used for three or five months and an injection that is used for three months*.’ Disadvantage was further compounded with adolescents with disabilities, who were 17% less likely to be able to name a method of contraception in the quantitative survey compared to peers without disabilities. This reflects a tendency for young people with disabilities to be more likely to be excluded from sexuality education due to lower rates of school enrolment as well as discriminatory attitudes that sexual and reproductive health issues do not equally concern persons with disabilities.

Our qualitative research also pointed to important inter-regional differences in gender norms governing contraceptive use. In South Gondar, Amhara, both married and unmarried girls use contraception; in marriage, it is used with the support of husbands to delay pregnancy and have better childbirth outcomes, and for unmarried girls, it is with family support due to fear of pregnancy out of marriage as a result of rape. An adolescent girl in South Gondar explained that even girls who do not have boyfriends ‘*get injected, fearing rape and getting pregnant*’. A father in the same community explained that he proactively encouraged his daughter use contraception: ‘*We have to teach the young girls to use contraceptives in order to become safe. I took my daughter to the health centre and made her use contraceptives. She has no [boy] friends but I did it for safety.*’

The qualitative interviews found that girls in the research sites that did use contraception primarily used injectables. Yet in East Hararghe, Oromia, where early marriage is increasing (often initiated by adolescents, against their family’s wishes), the qualitative research found that contraception is not used to delay first pregnancies, as girls wish to prove they are fertile. One 14-year-old married girl from this area explained, ‘*I am not using [family planning] now – before I have one child. If you stay without a child for a longer time, they will tell you, you are barren.’* In Afar, where *absuma* (compulsory cousin) marriage is practised, girls are increasingly using contraception to prevent pregnancy before marriage – often with boys’ agreement, as pregnancy out of marriage is heavily penalised.

In Afar and Amhara, the qualitative research also found that changes in adolescents’ sexual behaviour (especially increased engagement in premarital sex) is leading to more contraceptive uptake, but the most readily available and socially acceptable contraceptives at family planning clinics are the combined oral pill rather than condoms. Condom supplies are limited (particularly in rural areas), and their use is often stigmatised. A health extension worker in South Gondar noted ‘*a shortage of condoms in Debre Tabor town in particular and at the national level in general’;* another in East Hararghe underscored that supplies are insufficient to meet demand.

## Discussion

The qualitative and quantitative data presented here show the ways that gender intersects with other social identities and the broader context of adolescents’ lives to shape the outcomes and experiences of adolescents across four key areas of health. As they progress through adolescence, young people encounter myriad health-related challenges. Yet these should not be understood as individualised experiences; they are inherently relational and structural - especially in terms of the broader socioeconomic context in which they live - and are subject to change over time. Seeing inequalities in the health outcomes of adolescents must be understood not just as individual problems, but as intrinsically linked to broader social relations and practices. This means attending both to the role of peers, community workers, healthcare providers and family in adolescents’ health; and to how gender, age and other social identities may mediate their opportunities for building relationships that can provide them with information and support. An intersectional approach also draws attention to the ways that these relations of power marginalise and exclude certain groups within society [32].

Gender has historically been the focus of research with adolescents in Ethiopia in relation to health. Our research findings nuance understanding of gender inequalities in relation to health outcomes by showing that gender norms *intersect* with structural disadvantages (including poverty, distress migration and limited access to healthcare in remote rural areas) to generate different types of inequalities in adolescent girls’ and boys’ health outcomes. For example, whilst gender norms around what is socially acceptable for girls prevent them from engaging in substance abuse for example, adolescent girls experience other health challenges. A significant gender issue highlighted in these findings is the social barriers that exist to contraceptive access and use. In all geographic areas where research was undertaken, cultural norms and the value placed on motherhood make contraceptive use particularly difficult for younger women. This echoes findings elsewhere by Baird et al. on the relationship between gender norms and health outcomes [40].

Yet gender norms may not always manifest in the same way across specific locations. Captured in the above findings is the dynamic relationship between local cultural norms around sexuality and gender, and sexual and reproductive health provision - which may be overlooked or obscured in research which does not address the nuances of different contexts. In Afar, whilst condom use is stigmatised, the contraceptive pill is more readily available. Key to the changing norms around contraceptive use in Afar is *sadah*, a cultural dance where adolescents can interact with the opposite sex outside of parental supervision and surveillance. In the past, the risk of losing livestock (as compensation in the event of pregnancy outside of marriage) deterred sexual activity during *sadah*, but in recent years, adolescents and some district key informants noted that the practice has become more sexualised – made possible by the increased availability of contraception.

Focusing on these ‘mutually constitutive relations among social identities’ [31] generates a number of practical implications for improving practices aimed at addressing poor adolescent health in key areas. Whilst education is not a panacea to health inequalities, the lack of knowledge shown by certain cohorts’ underlines that there is a need for specific measures to include younger and rural adolescent girls in programming. This is particularly the case for information about puberty, as the data shows they are even less likely to be able to access the information they need about their bodies before and during puberty. Even where classes on menstruation in school do exist, they cannot be relied upon to introduce these ideas; they are only introduced at upper primary level, by which point many students will have already started puberty, and many others will have left school altogether (given current high dropout rates found) [41].

The findings around contraceptive knowledge have implications for sexual health strategies which effectively target young people. Concerns about HIV and limited access to services and supplies were widely reported by adult respondents in both rural and urban areas. Migration to cities from rural areas by adolescent boys produces new health challenges because of the exposure to risks that urban life generates, especially regarding substance use and traffic accidents. Several reasons for rising rates of HIV in Amhara and Oromia were cited in the qualitative research, including rural–urban migration having led to changes in relationship structures. Young men who move for work are engaging in risky sexual behaviour in urban areas and then returning home, unaware that they are spreading sexually transmitted illnesses. Migration for work is driven by poverty and lack of opportunities for young men in rural areas. Yet respondents often put the blame for infidelities on mistresses, illustrating the strength of gender norms around sexuality. Unequal health information and outcomes cannot therefore solely be addressed through expanding education and accessibility of health services.

Working from an intersectional perspective underlines the importance of considering young people not just as individuals at the intersection of different types of inequalities (such as gender, age, disability and location) but also as embedded within networks of relationships. Adolescence is not just a biological change, but a social experience. When it comes to menstrual hygiene management and puberty, for example, these relationships determine whether young people receive accurate and timely information. Yet the research shows that many caregivers, especially in rural areas, lack accurate knowledge and thus cannot advise adolescents, instead reproducing harmful and stigmatising ideas about gender and sexuality. Menstruation is sometimes associated with being sexually active; this puts off younger girls in particular from approaching caregivers to ask for information about puberty [42]. This means that efforts to promote education and awareness around puberty and menstrual hygiene need to engage with families.

Shifting attention away from individuals and towards social relations and practices which reproduce gender inequalities is also essential for interventions around sexual health via youth-friendly programming and peer education that have proliferated in recent years. There are signs of government initiative in this area. For example, Ethiopia’s National Adolescent and Youth Health Strategy (2016–2020) is the first of its kind to go beyond sexual and reproductive health to address ‘nutrition, mental health, substance use, non- communicable diseases, intentional and unintentional injuries, various forms of violence, and risks and vulnerabilities associated with disability’ [43]. The strategy also recognises that various aspects of young people’s lives may affect their access to healthcare. These include how their time is structured, their mobility outside of the home and their parents’ influence, all of which mediate their capacity to access such initiatives [14].

However, reconfiguring healthcare systems to make them more accessible to adolescents still requires a more nuanced understanding of what ‘accessibility’ really means [4]. While community health extension services in Ethiopia have improved preventive healthcare over the past 15 years, basic healthcare is still not consistently available in all areas of the country. The cadre of health extension workers which provide services for people in the most rural areas are a key means for delivering expanded services in relation to adolescents’ sexual and reproductive health needs - yet without adequate training and support, there is a risk that services will not be consistently ‘youth friendly’ and thus exclude adolescents. Indeed, health interventions which centre on gendered health issues like sexual health often take ‘gender’ to mean women; the specific needs of adolescent girls and younger women, who are often simply added into or included in gender programming as an afterthought, are not adequately considered and efforts to bring them in under this rubric can therefore fall flat [44]. An intersectional approach makes connections between gender, age and other forms of disadvantage and more fully engages with the ways in which they produce health inequalities.

### Study limitations

This study has a number of limitations which should be recognised and addressed in future research. Firstly, these baseline results can only show correlations and should be interpreted in that light. Second, despite the efforts of research assistants to develop positive and trusting relationships with participants, social desirability may have had an impact on responses, especially in the survey where answers could not be explored in such depth. Thirdly, in rural areas older adolescents were only interviewed qualitatively, meaning that comparisons cannot be made between age and urban/rural location in the same ways that we have been able to do for gender differences.

## Conclusion

Global health policy is increasingly recognising that adolescence offers a unique window in which to accelerate progress in tackling health inequalities. However, as the findings in this paper highlight adolescent transitions shape girls’ and boys’ lives in highly gendered ways, due to the prevailing norms in their socio-cultural environments. These norms – especially those that govern gender roles and sexuality – start to become more rigidly enforced and more consequential in early adolescence, forcing girls’ and boys’ trajectories to diverge as they approach adulthood. Tailoring interventions so that they are informed by this divergence will be key, and critical to achieving the 2030 Sustainable Development Agenda commitment to achieving health and wellbeing for all. Engaging with age and gender inequalities also demands nuanced attention to how adolescents’ experiences are shaped by broader social, cultural and economic dynamics, which change over time and as they age. An intersectional approach – which takes into account how these dynamics interact to enable and constrain adolescents’ capacities – is key to identifying opportunities to improve adolescent health interventions and ensure that the hardest-to-reach adolescents in LMICs are not inadvertently excluded from the information and services they need to ensure a healthy second decade of life and transition into adulthood.

## Figures and Tables

**Table 3:**
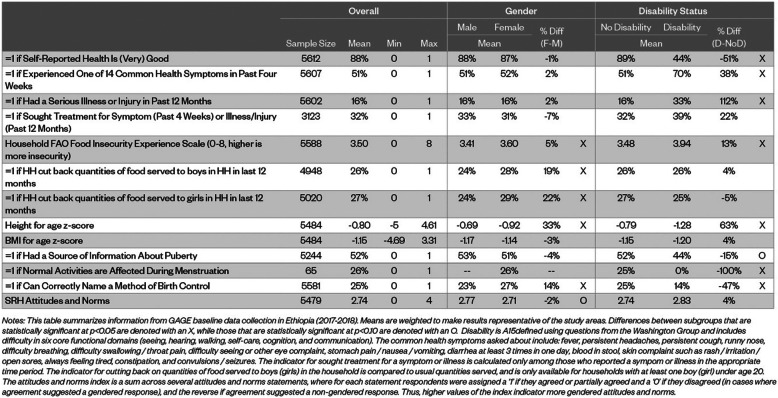
Health, Nutrition, SRH, Gender and Disability

**Table 4:**
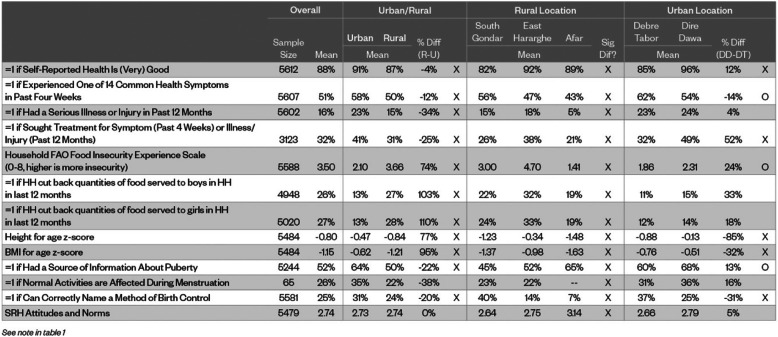
Health, Nutrition, SRH, Location

## Data Availability

The datasets produced and analysed during this study are available here: **SN 8597 - Gender and Adolescence: Global Evidence: Ethiopia Baseline, 2017–2018**https://beta.ukdataservice.ac.uk/datacatalogue/studies/study?id=8597

## Footnotes

1 *Khat* is a plant native to the Horn of Africa and contains a stimulant drug.
